# Feasibility of early rapid sequence induction and intubation training and the role of a cognitive aid on-demand reader in medical students: lessons from a pilot randomized study

**DOI:** 10.1016/j.bjane.2025.844701

**Published:** 2025-10-31

**Authors:** Getúlio Rodrigues de Oliveira Filho, Miguel Angelo Fabrin, Victor Medeiros Benincá, Ian Novy Quadri, Gabriel Resun Gomes da Silva

**Affiliations:** Universidade Federal de Santa Catarina, Departamento de Cirurgia, Florianópolis, SC, Brazil

Dear Editor,

Rapid Sequence Induction and Intubation (RSII) is a key part of safe airway management in emergency and perioperative care. Since its initial description by Stept and Safar in 1970,^1^ RSII has been recognized as a time-sensitive procedure that demands careful preparation, proper drug sequencing, and effective team coordination. In Brazil and other developing countries, newly graduated physicians often take on responsibilities for airway management in emergency departments immediately after medical school, often without specialized anesthesia training.^2^ This situation highlights the need for developing effective, scalable, and early educational strategies to ensure that future physicians can safely perform life-saving procedures, such as RSII. Recent evidence emphasizes the importance of preparing medical students with anesthesia-related skills to support the global surgery agenda and to reduce disparities in access to safe anesthesia care.^2^

We briefly report a pilot study aimed at assessing the feasibility of incorporating RSII training into the medical school curriculum shortly after students develop basic skills in tracheal intubation. Additionally, we sought to evaluate the potential impact and logistical considerations of providing a cognitive aid “reader” to assist students during simulation.^3^

We conducted a prospective, randomized simulation study with 44 medical students from the 3rd to 6th year of medical school. The institutional research ethics committee approved the study (Certificate of Presentation for Ethical Consideration [CAAE: 58,776,122.6.0000.0121]), and participants signed written informed consent before enrolling. Participants completed an online preparatory module covering indications for RSII, preoxygenation, pharmacology of induction agents and neuromuscular blockers, and a step-by-step checklist of 17 essential tasks. Students were then randomized to perform a standardized RSII scenario with or without the presence of a trained cognitive aid reader, who followed the checklist and was available to read steps upon the students’ prompts. Procedures were video-recorded, and performance was evaluated by blinded assessors using the 17-item checklist as the primary outcome. Secondary outcomes included adherence to the correct sequence, number of technical errors, time to completion, and overall performance rating.

The median number of checklist items completed was 17 (IQR 16–17) in the reader group and 16 (IQR 15–17) in the control group (*p* = 0.11). No statistically significant differences were observed between groups regarding adherence to the correct sequence, technical errors, or overall scenario duration. Reader activation occurred in approximately 22 % of steps, most commonly during drug administration and preparation for intubation. Both groups reported high satisfaction with the training and increased perceived preparedness for airway management.

In our study, having a cognitive aid reader did not significantly boost performance in a simulated RSII scenario. This indicates that combining structured preparatory materials with simulation exposure might be enough for students to perform at a high level early in their training. These findings match previous research showing that cognitive aids are more helpful in less structured, crisis-like situations or for participants facing high cognitive load, rather than for learners who have just undergone focused training.^4^

The study was conducted at a single institution with a relatively small sample size, which limits its generalizability. Students from various academic years were intentionally included, which may have introduced variability in baseline knowledge and confidence; however, this was not tested due to the small sample size. We used a medium-fidelity manikin, which cannot replicate all clinical cues of a real RSII; however, the choice reflects the reality of most medical schools in Brazil and other developing countries, where high-fidelity simulators are not routinely available. This makes our results more realistic for similar settings. Additionally, readers were instructed to follow the cognitive aid script exactly, simply reading each item as written, without changing timing or phrasing. This consistent approach ensured reliability but may have limited the flexibility of the intervention.

Despite these limitations, this pilot study demonstrates the feasibility of introducing RSII training soon after tracheal intubation instruction in the regular medical curriculum. The results also suggest that including a cognitive aid reader may not provide measurable benefits in this context, raising essential considerations about the logistics and cost-effectiveness of deploying readers as part of routine RSII training.

In conclusion, early RSII training is feasible and effective when supported by structured preparation and simulation-based education. The addition of a cognitive aid reader did not produce measurable performance improvement, indicating that resources might be better spent on ensuring high-quality pre-simulation preparation and structured debriefing.

Future studies should investigate whether cognitive aids become more relevant under stress conditions, with multiprofessional teams, or in more complex airway scenarios, and whether repeated exposure throughout medical school translates into improved clinical performance after graduation.

## Data availability statement

The datasets generated and/or analyzed during the current study are available from the corresponding author upon reasonable request.

## Declaration of AI use

During the preparation of this work, the authors used Grammarly and ChatGPT to improve grammar, clarity, readability, and aid in rephrasing sentences for conciseness and flow. After using these tools, the authors carefully reviewed and edited the content to ensure accuracy, scientific integrity, and compliance with journal guidelines, and they take full responsibility for the content of the published article.

## Authors’ contributions

Getúlio Rodrigues de Oliveira Filho helped with the study's conception, supervision, manuscript writing, and final version approval.

Miguel A. Fabrin helped with the study's conception, manuscript writing, and final version approval.

Victor M. Benincá helped with the study's conception and the approval of the final version of the manuscript.

Ian N. Quadri helped with the study's conception and the approval of the final version of the manuscript.

Gabriel R. G. da Silva helped with the study's conception and the approval of the final version of the manuscript.

## Conflicts of interest

The authors declare no conflicts of interest.
